# Impact of Sleep Duration, Quality, and Chronotype on Learning and Academic Performance: A Cross-Sectional Study Among First Year Medical Students of a Tertiary Care Institute

**DOI:** 10.7759/cureus.50413

**Published:** 2023-12-12

**Authors:** Sangeeta Gupta, Manoj Prithviraj, Anil Gangwar, Rama S Rath

**Affiliations:** 1 Physiology, All India Institute of Medical Sciences, Gorakhpur, Gorakhpur, IND; 2 Psychiatry, All India Institute of Medical Sciences, Gorakhpur, Gorakhpur, IND; 3 Community Medicine, All India Institute of Medical Sciences, Gorakhpur, Gorakhpur, IND

**Keywords:** epworth sleepiness scale, pittsburgh sleep quality index, morningness-eveningness questionnaire, sleep duration, sleep quality, learning, gpa, chronotype, academic performance

## Abstract

Introduction

The link between sleep and cognitive processes, such as memory and learning, continues to be one of the most intriguing and perplexing theories. Undergraduate medical students in their first year are particularly vulnerable to sleep disturbances. Academic achievement and learning have been linked to sleep patterns, which include not only the quantity and quality of sleep but also the timing of sleep in relation to the natural sleep onsets, or chronotypes. There have been conflicting reports on the outcomes of sleep and relatively fewer researches focused on the impact of chronotypes on learning and academic achievement among medical students. The current study thus sought to determine the chronotypes of medical students, evaluate the quantity and quality of sleep, and determine the impact of these factors on learning and academic performance.

Methods

The study was conducted in the Department of Physiology, All India Institute of Medical Sciences (AIIMS) Gorakhpur, India. Sleep health was assessed in 167 first-year medical students using the Pittsburgh Sleep Quality Index (PSQI), Epworth Sleepiness Scale (ESS), morningness-eveningness questionnaire (MEQ), and sleep log books. Learning and memory assessment was performed using Raven's progressive matrices test. Grade point average (GPA) was used to assess their academic performance. The relationship of sleep scores with GPA and RPM scores were obtained by linear regression analysis. One-way analysis of variance (ANOVA) and unpaired t-test were used to investigate other comparisons among categories of chronotypes and those of mean GPA. A p-value of <0.05 was considered as significant.

Results

The mean GPA and RPM scores obtained in the groups with PSQI ≥ 5 (2.67 ± 1.1, 49.51 ± 6.24, respectively) and PSQI < 5 (3.15 ± 0.59, 54.73 ± 4.01, respectively) and those in the group with ESS ≥ 10 (2.72 ± 1.17, 50.97 ± 5.92, respectively) and ESS < 10 (3.15 ± 0.6, 54.18 ± 3.91, respectively) varied with statistically significant differences (p < 0.05). Statistically significant R-squared values for the relationship of PSQI and ESS scores with RPM and GPA scores were obtained. No correlation between academic grades and chronotype was found. Poor GPA scores were found to be associated with reduced mean sleep duration for one week before the exams.

Conclusion

Learning and academic performance are negatively impacted by poor sleep quality and daytime sleep dysfunction. No definite evidence for the association of sleep chronotypes with the learning and memory could be attained. Higher test performance is more closely linked to the average sleep length over a duration of time preceding the exams.

## Introduction

The role of sleep in physical and mental health has been substantially demonstrated in the research [1-3]. However, the relationship of sleep with cognitive functions, including learning and memory, is still one of the most vexing and engrossing hypotheses. Not only the amount and the quality of sleep but also the sleep timings relative to the natural sleep onsets, the chronotypes, have been suggested to influence learning and academic achievements [4,5]. Chronotype is the circadian preference of the individual to the timings of sleep and wakefulness. Chronotype is predominantly controlled by the circadian clock and external timing signals (zeitgebers) [6]. Humans synchronize for 24 hours (entrainment), and individual variations in the endogenous timing system, i.e., circadian clock, give rise to the distribution of chronotypes, ranging from early (larks) or morning types to late (owls) or evening types [7]. Chronotype varies with age and is the latest during adolescence [7]. Chronotype is associated with academic performance along with other sleep features, including sleep quality, quantity, and regularity [8].

Although sleep disorders are prevalent in the general population as a whole, one of the subgroups that have been recognized as especially vulnerable to poor sleep is that of medical students. Greater academic load with long duration and high-intensity studies have been suggested as some of the important attributable factors [9]. Vulnerability to sleep disturbances has been particularly discerned in first-year medical undergraduates in various studies [10]. The first year of the study of medicine incorporates a phase of transition in which students undergo a change from the early schedules of preparatory courses for college entrance examinations and attending high schools to a different phase of undergraduate courses, which is characterized by plentiful academic activities, irregular daily routines, and a considerable increase in the academic load. Various cross-sectional studies indicate that evening circadian preferences, poor sleep quality, and inadequate sleep duration are associated with adverse outcomes, including worse academic performance and emotional stress among the students [11-13]. A few studies have interestingly found that the sleep quality, but not the sleep duration, of these students correlates with academic scores [14,15].

Despite a substantial amount of data on the outcomes of sleep among students, numerous studies offer conflicting or perhaps unexpected findings or have reported the effects as trivial [16-21]. Moreover, relatively little research has been done on the impact of chronotypes on learning and academic achievement among medical students. Investigating important sleep features can contribute to monitoring sleep health in students in their beginning year of the undergraduate course. Poor academic performance may lead to struggles and can cause significant stress. Identifying students at risk can help target them with programs to improve sleep and measures to impart general sleep education for medical students can be carried out. Furthermore, the influence of sleep observed on academic achievement is mediated by the effects of sleep on cognitive function, depending on the time of day when various chronotypes achieve their peak performance. Hence, the present study aimed at identifying the chronotypes of medical students to assess the sleep quality and quantity and to obtain their effects on learning and academic performances.

## Materials and methods

A analytical cross-sectional study was conducted involving 167 students at a tertiary care center from December 2020 to November 2022. The study was conducted in the Department of Physiology, All India Institute of Medical Sciences (AIIMS) Gorakhpur, India. The initial calculation of the sample size provided a figure of 120, after accounting for the prevalence of poor sleep quality as 77%, power to be 80%, error to be 0.05, and relative precision to be 10% based on a previous similar study [21]. However, with a non-response rate of 40%, a total sample size of 170 was obtained. 

Out of 170, data from three students could not be included (owing to inappropriately filled questionnaires/absence in the exam), so the study sample comprised 167 students. Approval for conducting the study was obtained from the institutional ethics committee (Institutional Human Ethics Committee (IHEC), All India Institute of Medical Sciences, Gorakhpur, India, reference number: IHEC/AIIMS-GKP/BMR/21/2020). Informed written consent was obtained before conducting the study.

The study comprised first-year medical students of AIIMS, Gorakhpur. Students were requested to voluntarily participate in the study. Students absent in the examination (included for academic assessment) and those who filled the questionnaires erroneously or in improper ways were excluded from the study. The presence of anxiety/depression or other psychiatric illnesses was another exclusion criterion for the study participants.

The students who provided the consent were asked to fill out the structured questionnaire, which included demographic information, e.g., gender, age, and cumulative attendance. Another section included a battery of self-administered questionnaires for the assessment of their sleep status. Chronotype was assessed by the morningness-eveningness questionnaire (MEQ). We used a validated reduced version of the original Horne and Ostberg morningness-eveningness questionnaire (MEQr) [22]. The MEQr originally established five behavioral categories: definitively morning-type (score, 22-25), moderately morning-type (score, 18-21), neither-type (score, 12-17), moderately evening-type (score, 8-11), and definitively evening-type (score, 4-7). In this study, we employed the simpler classification by Taillard and colleagues and other researchers: morning-type (M-type; score, 18-25), neither-type (N-type; score, 12-17), and evening-type (E-type; score, 4-11) [23].

Sleep quality was assessed by the Pittsburgh Sleep Quality Index (PSQI), a self-report questionnaire that assesses sleep quality over a one-month time interval and takes about five to 10 minutes to complete [24]. The measure consists of 19 individual items and seven components that produce one global score. Seven components, namely, subjective sleep quality, sleep latency, sleep duration, habitual sleep efficiency, sleep disturbances, use of sleeping medication, and daytime dysfunction, are analyzed in the PSQI. One global score is obtained by adding the scores for the seven elements, which range from 0 to 21, where lower scores denote a healthier sleep quality. A global PSQI score greater than 5 has been reported to yield a diagnostic sensitivity of 89.6% and specificity of 86.5% (p-value less than 0.001) in distinguishing good and poor sleepers [24]. The reliability and validity of the PSQI test have been reported to be acceptable [25].

The Epworth Sleepiness Scale was used to measure daytime sleepiness. The test consists of a list of eight circumstances in which the score of propensity to doze off on a scale from 0 (no likelihood of dozing off) to 3 (high chance of dozing off) is noted [26].

Another tool employed to assess the quantity of sleep for one week was a sleep log book/sleep diary (by the National Sleep Foundation) [27]. Sleep diaries are reliable instruments for collecting data about sleep/wake patterns in many previous studies [28]. A record of students’ sleeping and waking times with related information was obtained in the sleep diaries for the one-week duration in the current study.

Learning and memory assessment was performed using Raven's progressive matrices test (RPM). RPM (available from: https://psycho-tests.com/test/raven-matrixes-test), which is a nonverbal group test used often in educational settings, tests the reasoning abilities of participants. It is usually a 60-item test with increasing orders of difficulty levels. In each test item, the subject is asked to identify the missing element that completes a pattern. The test provides a score at the completion [29]. The raw score is simply the total number of items that the student answers correctly. For the standard version, as there are 60 items, the raw score could range from 0 to 60. In the current study, we analyzed the raw RPM test scores. Assessment of academic performance was done by calculating the grade point average (GPA) for all the three semester examinations, and the mean GPA was calculated for every student. 

The data were expressed as mean ± standard deviation (SD). PSQI, ESS, and MEQ scores were compared with the numerical value of mean GPA and RPM test scores by linear regression analysis. Comparisons of the mean GPA and RPM test scores among the groups were performed by unpaired t-test. Comparison of RPM test scores and GPA among different chronotypes was performed by one-way ANOVA. GPA was further subdivided into three groups (<3, 3-3.5, and >3.5), and the mean sleep duration in the three categories was compared by one-way ANOVA. A p-value of <0.05 was considered as significant. The analyses were performed in the IBM SPSS Statistics for Windows, version 28 (released 2021; IBM Corp., Armonk, New York, United States).

## Results

**Figure 1:**
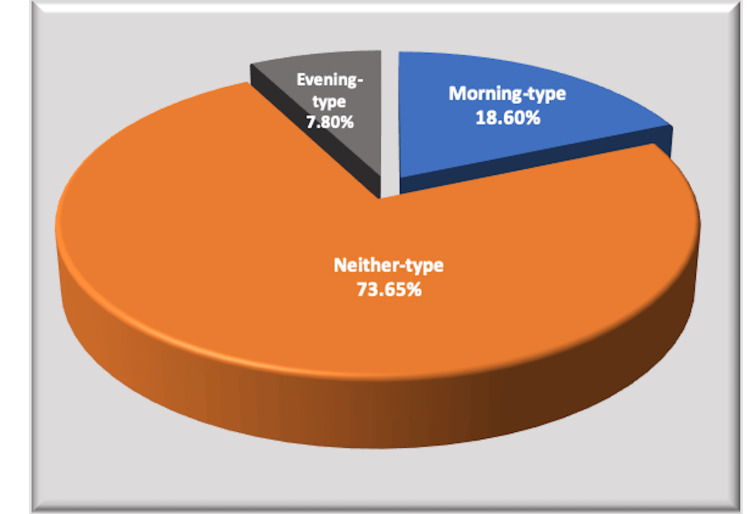
Percentage distribution of students with different chronotypes (by MEQr) MEQr: Morningness-eveningness questionnaire (reduced version)

The study included 167 students of first professional Bachelor of Medicine, Bachelor of Surgery (MBBS) with a mean age of 18.63 ± 0.45 years. Out of 167, 126 (75.44%) were males while 41 (24.5%) were females. The mean RPM test score (raw score) was 55.17 ± 5.02, while the mean GPA of the study participants was 3.02±1.22 (Table 1). Chronotype assessment by MEQr revealed the majority of the students (73.65%) are in the neither-type (N-type; score, 12-17) category, while morning-type students (M-type; score, 18-25) constituted 18.6%, and evening type (E-type; score, 4-11) were 7.80% (Figure 1).

**Table 1:** Study participants characteristics (n = 167) SD: standard deviation; GPA: grade point average; RPM: Raven’s progressive matrices; PSQI: Pittsburgh sleep quality index; ESS: Epworth sleepiness scale

Participant characteristics (n=167)	
Mean age	18.63 ± 0.45 (years ± SD)
Males (total number) (%)	126 (75.44%)
Females (total number) (%)	41 (24.5%)
GPA scores (mean ± SD)	3.02 ± 1.22
RPM test scores (mean ± SD)	55.17 ± 5.02
Poor sleep quality (PSQI score of ≥5) (total number) (%)	60 (35.93%)
Excessive daytime sleepiness (ESS score ≥ 10) (total number) (%)	41 (24.55%)

**Figure 2:**
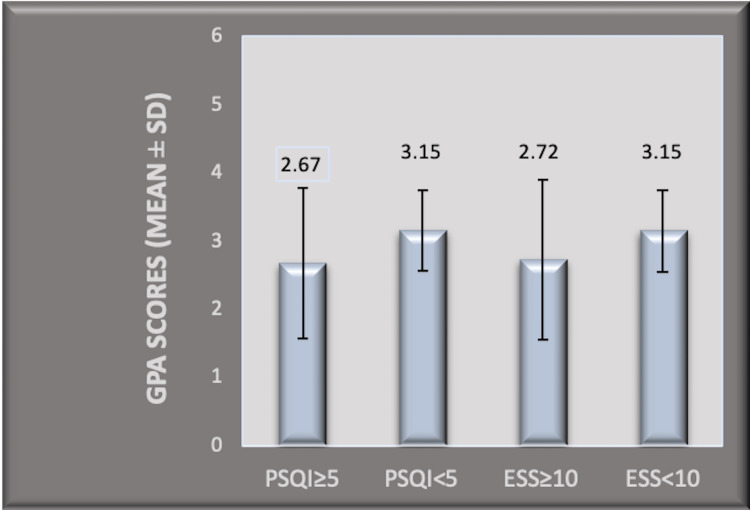
Comparison of mean GPA scores (mean ± SD) among the groups with PSQI ≥ 5, PSQI < 5, ESS ≥ 10, and ESS < 10. GPA: grade point average; SD: standard deviation;  PSQI: Pittsburgh sleep quality index; ESS: Epworth sleepiness scale

**Figure 3:**
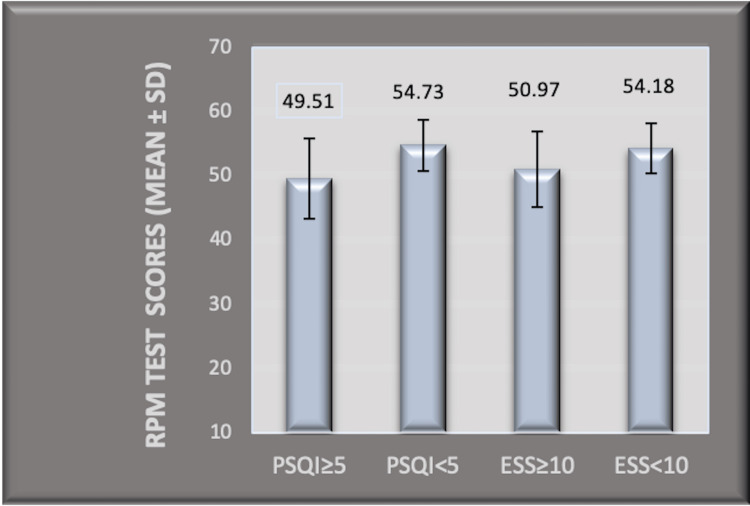
Comparison of RPM test scores (mean ± SD) among the groups with PSQI ≥ 5, PSQI < 5, ESS ≥ 10, and ESS < 10. RPM: Raven’s progressive matrices; SD: standard deviation; PSQI: Pittsburgh sleep quality index; ESS: Epworth sleepiness scale

Poor sleep quality with a PSQI score of ≥5 (significant sleep disturbance) was found in 60 students (35.93%) (Table 1). Their mean GPA was 2.67 ± 1.1. The mean GPA in students with a PSQI score <5 was 3.15 ± 0.59. The difference was statistically significant with p = 0.0008 (unpaired t-test) (Figure 2). The mean RPM test scores in the poor sleepers (PSQI ≥ 5) was 49.51 ± 6.24, while that with PSQI < 5 was 54.73 ± 4.01 (p < 0.0001) (unpaired t-test) (Figure 3).

Excessive daytime sleepiness with a total ESS score of ≥10 was obtained in 41 students (24.55%) (Table 1). The mean GPA in this group was 2.72 ± 1.17, while in the group with ESS score <10, it was 3.15 ± 0.6 (difference was statistically significant with a p-value of 0.0030) (unpaired t-test) (Figure 2). Mean RPM test scores in the group (ESS ≥ 10) was 50.97 ± 5.92, while that in the group with ESS score <10, it was 54.18 ± 3.91 (p-value = 0.0001, for the difference) (unpaired t-test) (Figure 3).

Linear regression analysis results for the relationship between sleep scores (PSQI, ESS, and MEQ) and RPM test scores and GPA scores were found to be statistically significant for PSQI and ESS scores, while MEQ scores could not be correlated with the RPM and GPA values with a statistically significant association (Table 2). Moreover, when the mean RPM test scores and mean GPA among the three chronotypes were compared with one-way ANOVA, the p-values were 0.95 and 0.53, respectively (Table 3).

**Table 2:** R-squared values for relationship of sleep scores (PSQI, ESS, and MEQ) with RPM test scores and GPA scores (dependent variables) by linear regression analysis * Significant (p < 0.05) PSQI: Pittsburgh Sleep Quality Index; ESS: Epworth Sleepiness Scale; MEQ: morningness-eveningness questionnaire; RPM: Ravens progressive matrices; GPA: grade point average

Variable	R-squared (R²)/ coefficient of determination		p-value	
	RPM test	GPA	RPM test	GPA
PSQI	0.20	0.07	<0.0001*	0.0005*
ESS	0.07	0.03	0.0004*	0.026*
MEQ	0.00017	0.0004	0.86	0.78

**Table 3:** Comparison of RPM test scores and GPA among different chronotypes by one-way ANOVA RPM: Ravens progressive matrices; GPA: grade point average; SD: standard deviation; ANOVA: analysis of variance

Chronotype	RPM test scores (mean ± SD)	p-value	GPA (mean ± SD)	p-value
Morning (n = 31)	53.67 ± 5.55	0.95	3.02 ± 0.15	0.53
Neither (n = 123)	52.98 ± 15.38		3.01 ± 0.27	
Evening (n = 13)	53.77 ± 14.21		3.09 ± 0.14	

The comparison among the three categories of GPA for the mean sleep duration (assessed by sleep logs) by one-way ANOVA yielded a p-value of 0.016 for the mean sleep duration for one week before the exams. Similar comparisons among the categories for the mean sleep durations for the nights just preceding the exams provided a p-value of 0.51 (Table 4).

**Table 4:** Comparison of sleep duration with GPA (one-way ANOVA) * Significant (p < 0.05) GPA: grade point average; hrs: hours; SD: standard deviation

GPA	Average sleep duration (one week preceding exams) (hrs) (mean ± SD)	p-value	Sleep duration (night just preceding exams) (hrs) (mean ± SD)	p-value
<3 (n = 28)	5.80 ± 1.2	0.016*	5.96 ± 1.84	0.51
3-3.5 (n = 126)	6.6 ± 1.48		6.38 ± 1.9	
>3.5 (n = 13)	6.9 ± 1.16		6.08 ± 1.34	

Gender and attendance were the two covariates tested in the relationship analysis. To find out whether gender was linearly related to the dependent variable (GPA), R-squared (R²) value and p-value were obtained by regression analysis (0.0003 and 0.81, respectively). The results indicated poor relationship between the variables (gender and GPA). When a similar analysis was performed for attendance as the covariate, the p-value was <0.05. Hence, it was added as a covariate in the general linear model for analysis. The p-value was 0.02 for the analysis performed. The p-value without the covariate (attendance) in the model (PSQI score vs. GPA) was, however, 0.0005. The null hypothesis could still be rejected, although with a smaller p-value when a covariate was added in analyzing the effect of sleep on the academic performance of the students.

## Discussion

Greater susceptibility to sleep disturbances has particularly been observed in the beginning year of medical students. Research on sleep disturbances in undergraduate medical students is of particular interest because of the known relationship between sleep and mental health and the concern that the academic demands of medical training can cause significant stress. Medical students go through long and intensive academic years before becoming physicians. Studies in the past have reported that first- and second-year students were particularly affected because of their worse subjective sleep quality [12,30]. Various factors, including medical students’ attitudes, knowledge of sleep, and academic demands, have been recognized as the causative factors [5]. Sleep deprivation has even been linked to depression, suicide, and a significant likelihood of substance abuse among adolescents [20]. Evaluating the quality of a student's sleep during the first year of an undergraduate course can be helpful in looking into significant sleep aspects.

In the current study, poor sleep quality (PSQI ≥ 5) was found in 35.93% of the students, which is a relatively smaller percentage in contrast with previous similar studies [31-33]. Most of the above-mentioned research included various phases of medical courses contrary to ours. Nonetheless, the proportion observed in our study remains consistent with a few prior such research from our nation and abroad [10,34,35]. Poor sleep quality obtained in first-year undergraduates has been well explained based on poor sleep hygiene habits, such as Internet surfing at night, poor social life, and bad eating habits [35].

Similarly, the proportion of students with daytime dysfunction found in the present study is consistent with the previous literature [36,37]. However, there are variations across studies in the proportion of medical students reporting daytime sleepiness [12,38].

Poor GPA and RPM scores obtained in the groups with poor sleepers ((PSQI ≥ 5) and that with excessive daytime sleepiness scores (≥10)) were statistically significant (p < 0.05) (Figures 2 and 3) in our study. Furthermore, statistically significant R-squared values for PSQI and ESS scores for learning and academic performances obtained in the study support the above findings (Table 2). Cognition has been reported to be influenced by insufficient sleep among medical students in the previous literature similar to our findings [39]. This finding lines up with the evidence from the past, stating the positive effect of sleep on memory, learning, acquisition of skills, and knowledge extraction [40,41]. Moreover, poor GPA scores reflecting poor academic performances, found in poor sleepers and those with daytime dysfunction, concord with previous reports among medical students [12,14,15].

The beneficial effect of sleep on learning and memory has been explained based on two main theories proposed in the past. The active system consolidation theory states that during sleep, new memories are reactivated and reorganized on a systemic level, with certain neural representations being potentiated and consequently strengthened [42-44]. Conversely, the synaptic homeostasis theory postulates that during sleep, synaptic connections undergo widespread depotentiation, with certain memory representations either experiencing less depotentiation or being spared from depotentiation, resulting in comparatively stronger memories [45,46]. There are experimental data to support both views, so they are not mutually exclusive. 

The chronotype of the students and its effects have received less focus than other aspects of sleep. Ideal sleep/wake times for college students are often two to three hours later; nevertheless, the class start times frequently remain the same. Previous reports have stated that the morning chronotype has a potential for improved academic accomplishment over the evening chronotype. Morning chronotypes considerably outperformed evening types on exams in the early and late morning, according to studies by van der Vinne et al. (2015), Randler and Frech (2009), and Zerbini et al. (2017) [47-49]. Interestingly, it has been reported in the past that crystallized intelligence (e.g., tasks of vocabulary and general knowledge) was not found to be influenced by MEQ scores, while when fluid intelligence (problem-solving, logic, and reason) was assessed and the individual completed those tasks during off-hours, i.e., when an individual with an evening preference took a test in the early morning, scores were significantly lower when all the other factors were held constant [47,49]. RPM test scores (which assessed fluid intelligence) were found to be the greatest (53.77 ± 14.21) in evening-type students in our study (Table 3). This group also exhibited the greatest mean GPA score (3.09 ± 0.14) (Table 3). However, the difference was not found to be statistically significant. Genzel et al. and Lai et al. also discovered no correlations between academic grades and chronotype confirming the present findings [50,51]. 

Poor GPA scores were found to be associated with reduced mean sleep duration (mean duration for one week before the exams) (Table 4). Sleep duration has been substantially reported to influence academic performances in previous reports [12,13]. However, in the present study, no significant association was obtained for the sleep duration for the night preceding the exams (Table 4) unlike prior studies [52,53].

Instead, we found that longer sleep duration over the week before the test was more associated with better test performance. These findings suggest that sleep has a critical function during the learning process of the content; restful sleep the night before might not be as beneficial. Prior research on sleep's role in memory consolidation supports the conclusion that more "content-relevant sleep" results in greater performance [44].

Limitations of the study

First, as it was a one-time cross-sectional survey, this study design does not permit the determination of a cause-and-effect relationship. Second, as the study was only carried out at one institution, it will be difficult to extrapolate findings from this study to medical students at other similar institutions.

The study consisted of virtually ethnically homogenous tertiary students (majority) from one geographical area, and thus our results might not apply to other multi-ethnic tertiary students in other parts. 

Lastly, measurement scales, such as the PSQI, ESS, and MEQ, are not accurate clinical diagnostic tools for sleeping disorders but are only subjective measures of sleep.

## Conclusions

Poor sleep quality and daytime sleep dysfunction impair learning and academic performance in first-year medical undergraduates. The average sleep duration over a period of time before exams may be more important for improved performance. The "neither-type" was the predominant chronotype found in the study. No significant association of chronotype with cumulative grade (GPA) was obtained.

A sleep evaluation of a student during their first year can be useful for exploring significant components of sleep and identifying students at risk. Sleep improvement programs should be encouraged and general sleep education should be tailored to the students susceptible to poor sleep health.
